# Global burden, trends, and projections of tetanus for 2050: Based on the 2021 Global Burden of Disease Study

**DOI:** 10.1016/j.cjtee.2026.01.001

**Published:** 2026-01-30

**Authors:** Tongying Liu, Menglong Liu, Guosheng Yang, Qingqing Ye, Tianjing Sun, Xiaojun Zhang, Anping Liu, Renjie Song, Can Luo, Xiaofei Huang, Anyong Yu, Haizhen Duan

**Affiliations:** a Emergency Department, The Affiliated Hospital of Zunyi Medical University, Zunyi, 563003, Guizhou Province, China; b Zunyi Medical University, Zunyi, 563003, Guizhou Province, China

**Keywords:** Tetanus, Disease burden, Epidemiological study, Decomposition analysis, Socio-Demographic Index, Bayesian age-period-cohort model

## Abstract

**Purpose:**

This study explores long-term trends and epidemiological characteristics of the global burden of tetanus over the past 3 decades and projects its disease burden up to 2050, providing a comprehensive assessment of the global impact of tetanus.

**Methods:**

The data of this study cover the period from 1990 to 2021, with projections made for 2050. Drawing on methodologies and findings of the Global Burden of Disease and Risk Factors Study 2021, employing key indicators such as prevalence, incidence, mortality, disability-adjusted life years, years lived with disability, and years of life lost provides a detailed account of the global burden of tetanus. By utilizing the socio-demographic index (SDI) values across different global regions, along with decomposition analysis and the Bayesian Age-Period-Cohort model, this study further elucidated the epidemiological patterns of tetanus and projected its global disease burden up to 2050. Integrating sample data and prior knowledge of unknown parameters yields posterior distributions for further parameter inference. This study uses the Bayesian Age-Period-Cohort and Integrated Nested Laplace Approximations packages in R for prediction and employs decomposition analysis to identify key factors influencing tetanus burden.

**Results:**

From 1990 to 2021, the global burden of tetanus showed a declining trend. In 2021, the disease burden was mainly concentrated among males and newborns. Specifically, the global incidence rate among males was 20,490 (95% uncertainty interval: 15,059–26,547), and among newborns was 14,263 (95% uncertainty interval: 5767–25,942). Within regions with different SDI levels, high-SDI regions had a lower disease burden, while low-SDI regions faced a heavier burden. Decomposition analysis indicated that epidemiological factors were the key determinants of the global burden of tetanus. The Bayesian Age-Period-Cohort projections suggested that by 2050, the global burden of tetanus would continue to decline, but the uncertainty interval would widen.

**Conclusion:**

The reduction in the global disease burden of tetanus reflects the success of global prevention and control efforts. Future prevention strategies should, building on existing immunization foundations, focus on newborns and men as key target groups, strengthen tetanus immunization for pregnant women, and intensify public awareness campaigns on tetanus prevention to maintain the disease burden at a low level.

## 1 Introduction

Tetanus, preventable by vaccination, is caused by a neurotoxin from *Clostridium tetani*. Its spores, widespread globally, easily contaminate wounds, even umbilical stumps in neonates, and develop into bacteria expressing toxin in anaerobic conditions, triggering the disease.^1^^,^^2^

Previously, the World Health Organization (WHO) had planned to completely eradicate tetanus by 1995. Attributed to the widespread promotion of high vaccination coverage, the incidence of tetanus has declined in most regions around the world. However, we still face numerous challenges in diagnosis and treatment.^3^, ^4^, ^5^ Relevant data indicate that the estimated number of global deaths caused by tetanus in 2015 was 34,019, representing a decrease compared to the annual death toll of approximately 800,000 cases in 1980. Notably, many studies tend to focus solely on specific regions or populations, lacking a comprehensive analysis from a global perspective. Taking 1993 as an example, the annual number of deaths due to maternal tetanus ranged from 15,000 to 30,000,^6^ highlighting the severity of the global tetanus issue. Upon further analysis of the 2015 data, we found that 79% of tetanus deaths (44,612 out of 56,743 cases) were concentrated in South Asia and sub-Saharan Africa, indicating that tetanus remains endemic in developing countries.^7^ Moreover, despite the WHO's recommendation since 1974 for the vaccination of all children with tetanus toxoid, there is still a relative scarcity of epidemiological research on neonatal and childhood tetanus globally.

Due to the limitations and challenges, there is an urgent need for a more comprehensive study on the global burden of tetanus. Researchers have utilized data from the Global Burden of Disease and Risk Factors Study 2021 (GBD 2021). This study encompasses health statistics from over 200 countries and has meticulously revised the estimation across the entire time series by continuously updating the latest data and research methodologies.^8^ Based on the authoritative data from GBD 2021, this study aims to comprehensively describe the global prevalence of tetanus by analyzing long-term trends in the tetanus burden from 1990 to 2021 and predicting its global prevalence up to 2050. We anticipate that this study will provide epidemiological evidence for health authorities to facilitate the further advancement of global tetanus prevention and control efforts.

## 2 Methods

### 2.1 Data sources

All data were publicly obtained from the website of the Institute for Health Metrics and Evaluation at the University of Washington (http://ghdx.healthdata.org/gbd-results-tool). The data on this website are primarily provided by international organizations and agencies (such as the WHO, the World Bank, and the United Nations Children's Fund), national health departments, academic research institutions, medical facilities, and healthcare providers. GBD 2021 integrated information from 204 countries and regions, covering 371 diseases and injuries, and systematically evaluated the epidemiological impacts of 87 risk factors.^9^^,^^10^ The epidemiological outcomes for diseases and injuries were estimated using multidimensional complex models, including the cause of death ensemble model, spatiotemporal Gaussian process regression, and the Bayesian meta-regression tool DisMod-MR.^9^ These models were employed to estimate key indicators such as prevalence, incidence, deaths, disability-adjusted life years (DALYs), years lived with disability (YLDs), and years of life lost (YLLs).^9^ In this analysis, the Global Burden of Disease Study Results Tool was utilized to extract global tetanus disease burden data from 1990 to 2021, including age-standardized rates and other comprehensive indicators. Additionally, the socio-demographic index (SDI) was extracted to further analyze the disease burden. This index is used to determine the position of countries or other geographical regions on the development spectrum. It ranges from 0 to 100, with 0 representing the lowest SDI value and 100 representing the highest. The SDI is calculated based on 3 indicators: lag-distributed income per capita, average years of schooling for individuals aged ≥15 years, and the total fertility rate among women under 25 years. Since only publicly available anonymized data were used, ethical approval was not required.

### 2.2 Case definition

In GBD 2021, tetanus is defined as a disease of skeletal muscle fibers caused by infection with the gram-positive bacterium *Clostridium tetani*. The disease is characterized by muscle spasms. The mode of transmission occurs through direct contact of *Clostridium tetani* with open wounds. The corresponding codes for tetanus in the International Classification of Diseases, 11th revision, are 1C13, 1C14, 8D83, 1C15NE60, QA08.2, PB28, PC98, PH48, PE88, PL00, QC03.1, QC03.2, and QC03.3. Among these, 1C13 refers to non-obstetric and non-neonatal tetanus, 1C14 refers to obstetric tetanus, 1C15 refers to neonatal tetanus, and the other codes pertain to tetanus vaccines and related harms associated with tetanus vaccines.

We analyzed the prevalence, incidence, mortality, DALYs, YLDs, and YLLs of tetanus globally and across regions in 2021 to evaluate the overall disease burden of tetanus. DALYs represent the sum of YLLs and YLDs.^11^ One DALY is equivalent to the loss of 1 year of healthy life. YLDs denote the number of years lived with any short- or long-term health impairment. They are calculated by multiplying the prevalence of a specific health condition by its corresponding disability weight. Disability weights are determined through public surveys, reflecting the severity of different health conditions. YLLs represent the years of life lost due to premature death and are calculated by subtracting the actual age at death from the highest possible life expectancy for individuals of that age. All estimates are accompanied by 95% uncertainty intervals (*UIs*), derived from 500 Monte Carlo simulations.^11^^,^^12^

### 2.3 Estimation of disease burden

We analyzed the disease burden of tetanus across different regions globally using varying SDI values to understand the impact of socioeconomic status on disease burden and reveal health inequalities. Disease burden data for 204 countries worldwide were obtained from GBD 2021 (http://ghdx.healthdata.org/gbd-results-tool), along with SDI values for these countries. Scatter plots with LOESS fitting lines were created using R software (version 4.5.1) to visually illustrate the impact of SDI on disease burden. Decomposition analysis was employed to dissect the disease burden of tetanus, quantify inequalities, and evaluate the effectiveness of interventions. Using the Das Gupta decomposition, changes in disease burden were decomposed into 3 indicators: epidemiological factors, aging, and population change. Epidemiological factors refer to the impact of disease risk factors (such as prevalence, incidence, and mortality) and related interventions on disease burden, excluding demographic factors. Aging denotes the impact of the elderly population on disease burden, while population change refers to the impact of the absolute total population size on disease burden. The contribution value (absolute effect) and contribution ratio (relative effect) of each factor to the overall change were calculated separately. The results were presented in graphical form to visually demonstrate the impact of each factor on health trends. The Bayesian Age-Period-Cohort (BAPC) analysis model was used to predict the future disease burden of tetanus globally. This model employs the Integrated Nested Laplace Approximation method to approximate marginal posterior distributions. This approach effectively addresses common mixing and convergence issues in Markov Chain Monte Carlo techniques while maintaining high computational efficiency.^13^ The BAPC model has been extensively validated and applied in epidemiological research due to its robust ability to capture comprehensive time trends, particularly in analyzing age-structured data and complex cohort effects.^14^ By integrating prior information with sample data, the model estimates posterior distributions and infers unknown parameters, generating accurate and well-calibrated probabilistic forecasts.^15^ In this study, posterior distributions were estimated by combining sample data with prior knowledge of unknown parameters, which were then inferred. Statistical analysis and visualization were performed using R software (version 4.5.1). Decomposition analysis and BAPC analysis were conducted in the R environment to identify the driving factors behind the data and predict future trends in disease burden.

## 3 Result

### 3.1 Overview

From 1990 to 2021, there was an overall declining trend in the global disease burden of tetanus, covering both the number of cases across all age groups and the age-standardized rate per 100,000 population, as shown in Table 1. Males show a higher relative reduction in cases compared to females. Simultaneously, the global age-standardized rate of tetanus per 100,000 population also declined, and the prevalence rate was higher among males than among females. Between 1990 and 2021, the total number of cases and the age-standardized rate per 100,000 population of tetanus prevalence across all age groups declined in regions with different SDI levels, though the extent of the decline varied. In terms of SDI levels and the prevalence rate per 100,000 population across regions, high-SDI regions saw declines in both aspects. High-middle and middle-SDI regions recorded significant decreases, while low-middle SDI areas also experienced declines. Although low-SDI regions showed reductions, they had the lowest decline among all regions.

**Table 1:** Number of tetanus prevalence cases and age-standardized prevalence rates of tetanus across global regions in 1990 and 2021 (values in parentheses represent 95% uncertainty intervals).

	All-age cases						Age-standardized rates per 100,000 people					
	Both		Male		Female		Both		Male		Female	
	1990	2021	1990	2021	1990	2021	1990	2021	1990	2021	1990	2021
Global	99,620 (75,905–125,498)	37,246 (27,879–48,218)	57,058 (42,688–71,389)	20,490 (15,059–26,547)	42,562 (32,841,54,106)	16,757 (12,678–21,395)	1.80 (1.37–2.27)	0.48 (0.36–0.62)	2.04 (1.53–2.57)	0.53 (0.39–0.68)	1.55 (1.19–1.97)	0.44 (0.33–0.56)
High-SDI	641 (477–840)	215 (170–278)	399 (295–523)	133 (104–171)	241 (181–322)	82 (64–108)	0.07 (0.06–0.10)	0.02 (0.02–0.03)	0.09 (0.07–0.12)	0.03 (0.02–0.03)	0.06 (0.04–0.07)	0.02 (0.01–0.02)
High-middle SDI	8729 (6452–11,201)	1796 (1285–2407)	5504 (4035–7090)	1009 (706–1383)	3224 (2359–4202)	787 (580–1046)	0.82 (0.61–1.05)	0.14 (0.10–0.19)	1.04 (0.76–1.34)	0.16 (0.11–0.21)	0.61 (0.45–0.8)	0.13 (0.10–0.17)
Middle SDI	34,592 (26,079–44,266)	9864 (7508–12,839)	19,286 (14,461–24,583)	4959 (3713–6422)	15,306 (11,567–19,683)	4905 (3769–6396)	2.01 (1.52–2.57)	0.41 (0.31–0.53)	2.20 (1.66–2.82)	0.41 (0.30–0.52)	1.8 (1.36–2.31)	0.41 (0.32–0.54)
Low-middle SDI	40,346 (30,827–50,446)	15,636 (11,766–20,127)	22,941 (17,020–28,312)	8574 (6332–11,049)	17,405 (13,465–22,087)	7062 (5295–9045)	3.27 (2.5–4.13)	0.81 (0.61–1.05)	3.64 (2.69–4.56)	0.89 (0.65–1.14)	2.88 (2.21–3.64)	0.74 (0.55–0.95)
Low SDI	15,273 (11,746–19,742)	9716 (7075–12,531)	8907 (6661–11,700)	5805 (4233–7535)	6365 (4712–8470)	3911 (2849–5124)	2.77 (2.12–3.48)	0.86 (0.63–1.11)	3.25 (2.45–4.16)	1.04 (0.75–1.35)	2.29 (1.71–3.02)	0.69 (0.5–0.91)
Central Europe, eastern Europe, and central Asia	13 (9–17)	6 (4–9)	27 (19–35)	3 (2–4)	40 (29–52)	3 (2–5)	0.01 (0.01–0.01)	0 (0–0)	0.01 (0.01–0.01)	0 (0–0)	0.01 (0.01–0.01)	0 (0–0)
High-income	128 (100–159)	27 (17–40)	64 (50–81)	15 (9–23)	64 (50–78)	12 (8–18)	0.01 (0.01–0.02)	0 (0–0)	0.01 (0.01–0.02)	0 (0–0)	0.01 (0.01–0.02)	0 (0–0)
Latin America and Caribbean	1086 (814–1424)	302 (185–458)	690 (518–900)	192 (121–285)	396 (295–530)	111 (64–178)	0.28 (0.21–0.37)	0.05 (0.03–0.08)	0.36 (0.27–0.47)	0.07 (0.04–0.10)	0.2 (0.15–0.27)	0.04 (0.02–0.06)
North Africa and Middle East	3153 (2291–4145)	2296 (1738–2951)	1886 (1329–2582)	1394 (1034–1790)	1268 (913–1709)	902 (683–1162)	0.82 (0.61–1.07)	0.37 (0.28–0.47)	0.96 (0.69–1.25)	0.43 (0.32–0.55)	0.68 (0.49–0.92)	0.30 (0.23–0.38)
Southeast Asia, East Asia, and Oceania	41,447 (31,344–53,048)	10,717 (8014–14,005)	23,128 (17,318–29,800)	5166 (3756–6753)	18,318 (13,781–23,467)	5552 (4213–7225)	2.47 (1.86–3.16)	0.51 (0.38–0.67)	2.72 (2.02–3.52)	0.48 (0.35–0.63)	2.22 (1.67–2.84)	0.55 (0.42–0.71)
Sub-Sahara Africa	10,478 (7694–1,4127)	8337 (6038–1,0779)	5840 (4264–7940)	4943 (3538–6480)	4638 (3248–6416)	3394 (2439–4481)	2.02 (1.50–2.61)	0.73 (0.53–0.94)	2.35 (1.73–3.05)	0.89 (0.63–1.17)	1.71 (1.20–2.32)	0.57 (0.41–0.76)

SDI: socio-demographic index.

Additionally, regions such as Central Europe, Eastern Europe, and Central Asia, and high-income regions had a relatively low disease burden, while South Asia, East Asia, and the Pacific regions still bore a heavy disease burden (Table 1). Furthermore, the global burden of tetanus exhibited similar trends in terms of incidence, mortality, DALYs, YLDs, and YLLs, as shown in Supplementary Materials 1–5.

Fig. 1A–F illustrates the global number of tetanus cases, incidence, deaths, DALYs, YLDs, and YLLs across all age groups in 1990. As can be seen from the figure, tetanus was highly prevalent among newborns under 28 days old and individuals aged 5–60 years, with a higher incidence in males than in females. By 2021, the figure demonstrates a decline in the global burden of tetanus. However, newborns under 28 days old still represented the age group with the highest burden of tetanus disease.

**Fig. 1:**
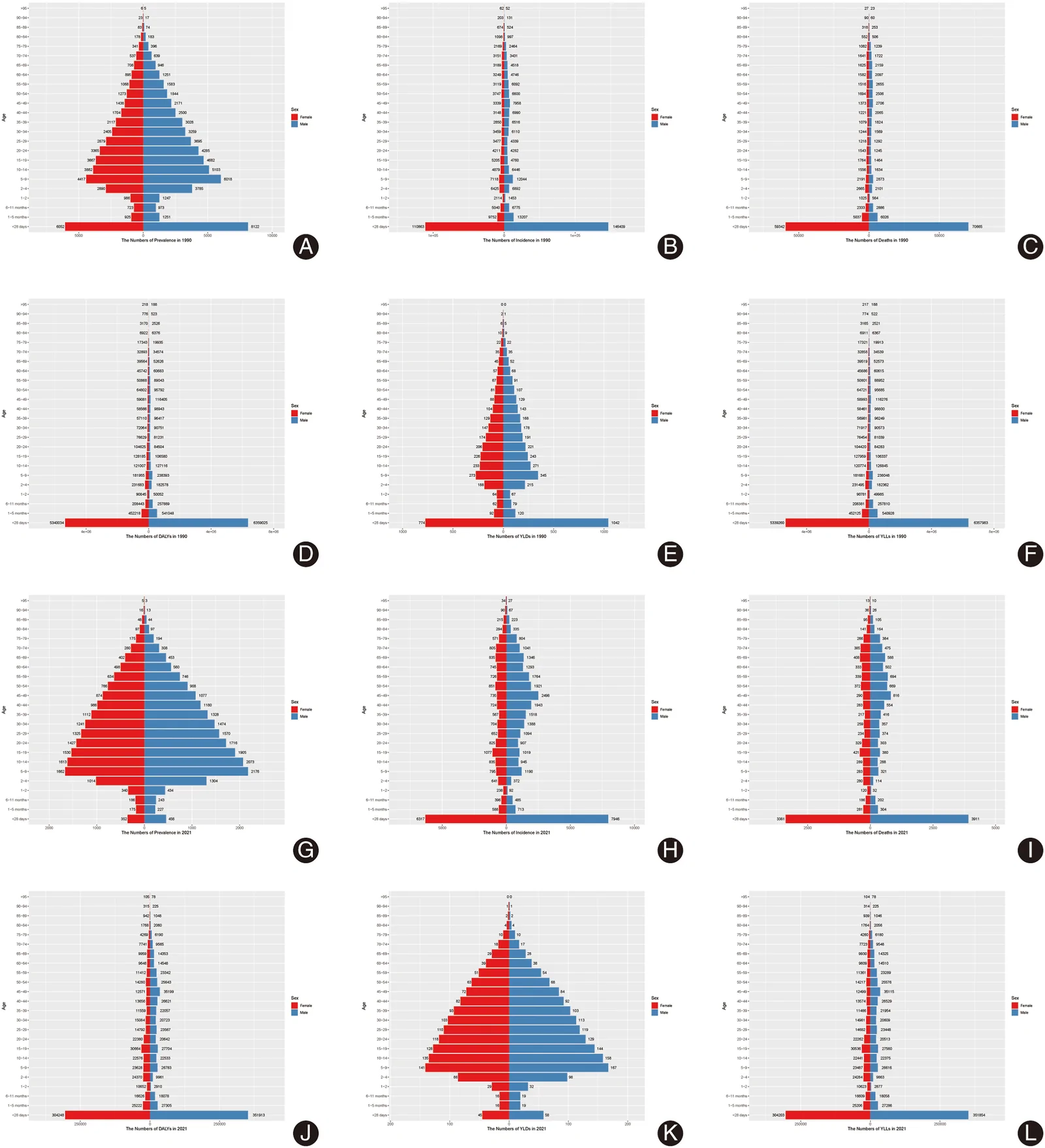
Global burden of tetanus disease age-sex pyramid in 1990 (A–F) and 2021 (G–L). DALYs: disability-adjusted life years; YLDs: years lived with disability; YLLs: years of life lost.

As shown in Fig. 2, the analysis of global temporal trends in tetanus reveals a continuous decline in the number of cases, incidence, deaths, DALYs, YLDs, and YLLs. The disease burden is slightly higher among males than among females (left vertical axis in Fig. 2A–F, bar charts). Similarly, the age-standardized rate per 100,000 population also shows a downward trend, with males bearing a slightly higher burden than females (right vertical axis in Fig. 2A–F, line graphs). By 2021, Fig. 2 illustrates the global distribution of tetanus cases, incidence, deaths, DALYs, YLDs, and YLLs by age groups. Newborns under 28 days exhibit higher numbers of cases, deaths, DALYs, and YLLs. In terms of the number of cases and YLDs, both newborns under 28 days old and individuals aged 5–70 years show relatively high values (left vertical axis in Fig. 2G–L, bar charts). Regarding the age-standardized rate per 100,000 population, newborns under 28 days old have a higher disease burden rate (right vertical axis in Fig. 2G–L, line graphs).

**Fig. 2:**
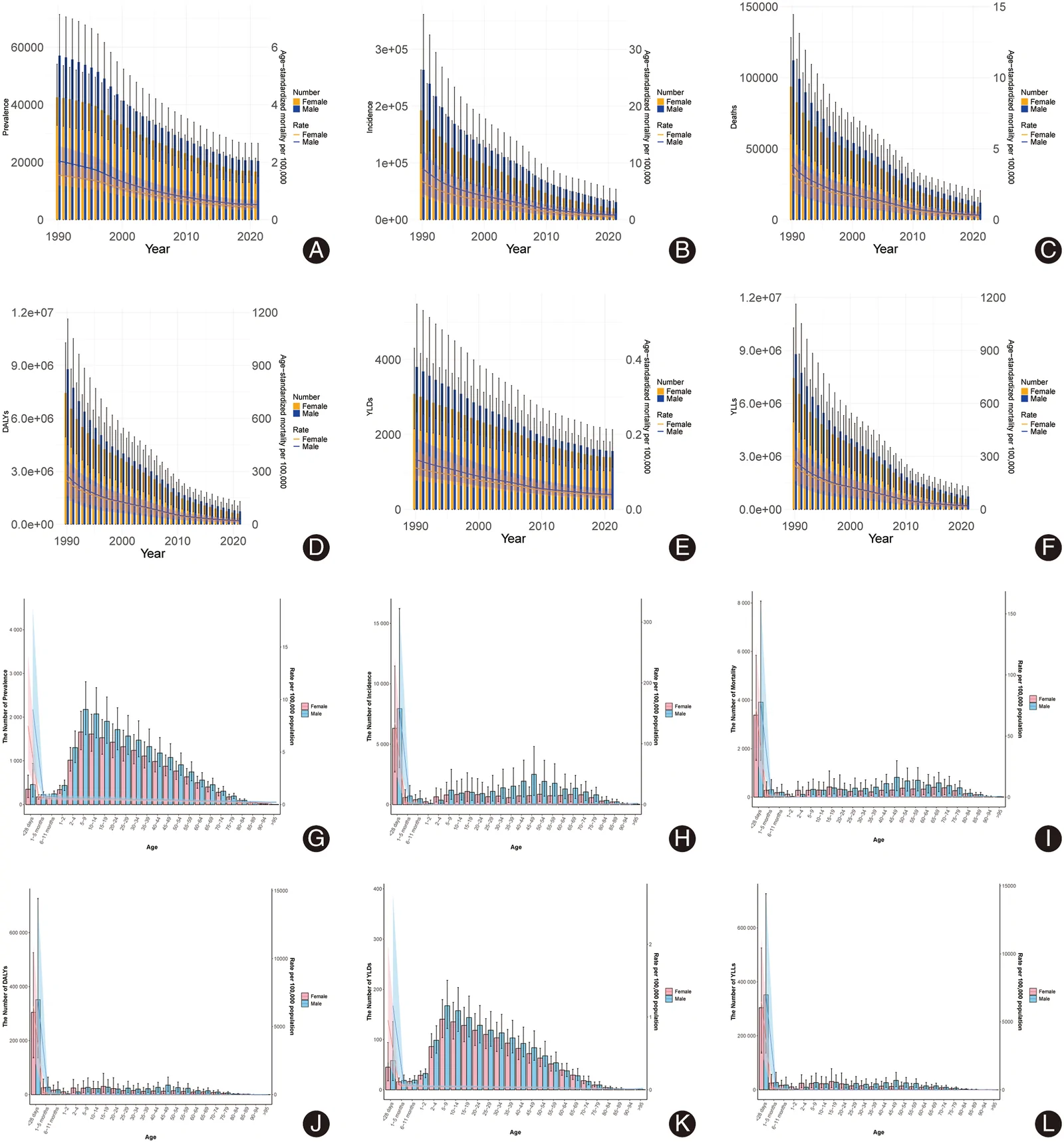
Temporal trends (1990–2021) and age distribution (2021) of global tetanus disease burden. (A–F) Show trends over time; (G–H) depict burden across age groups in 2021. DALYs: disability-adjusted life years; YLDs: years lived with disability; YLLs: years of life lost.

### 3.2 Analysis of disease burden across different SDI regions

By 2021, the burden of tetanus across various indicators had approached relatively low levels. Among them, high-SDI regions, high-middle-SDI regions, middle-SDI regions, and low-middle-SDI regions experienced significant declines, whereas the low-SDI region showed a relatively gentler decrease compared to other regions. In 2021, the disease burden in low-SDI regions remained higher than that in other regions, as shown in Fig. 3. By analyzing the disease burden across 204 countries based on their SDI values, Fig. 4 reveals that Somalia had the highest disease burden. In most countries, the burden of tetanus decreased as the SDI value increased; however, in some countries, there was still a divergence from the trend line in terms of prevalence and YLDs as the SDI value rose (Fig. 4).

**Fig. 3:**
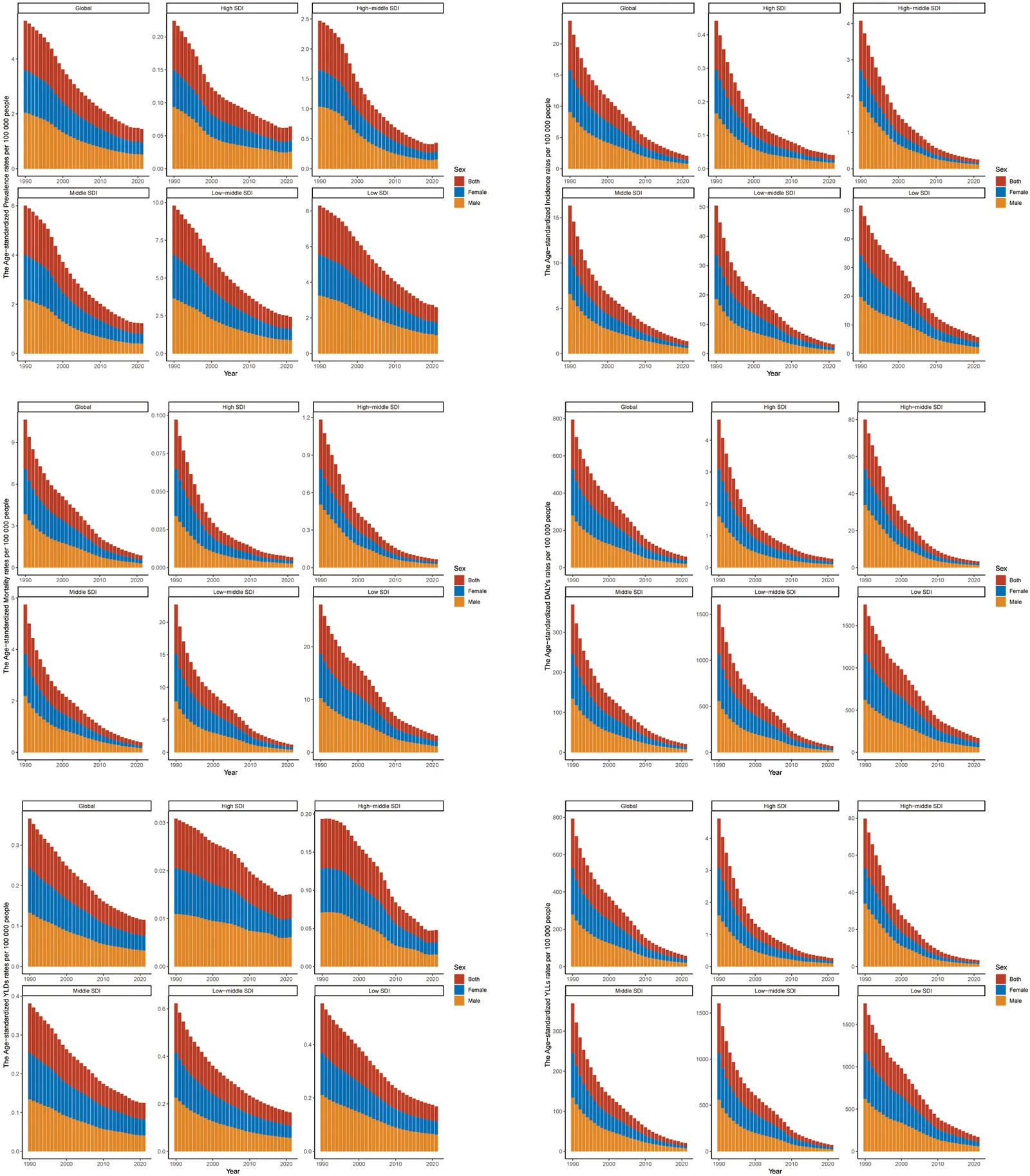
Analysis of disease burden across different SDI regions (per 100,000 population). SDI: socio-demographic index; DALYs: disability-adjusted life years; YLDs: years lived with disability; YLLs: years of life lost.

**Fig. 4:**
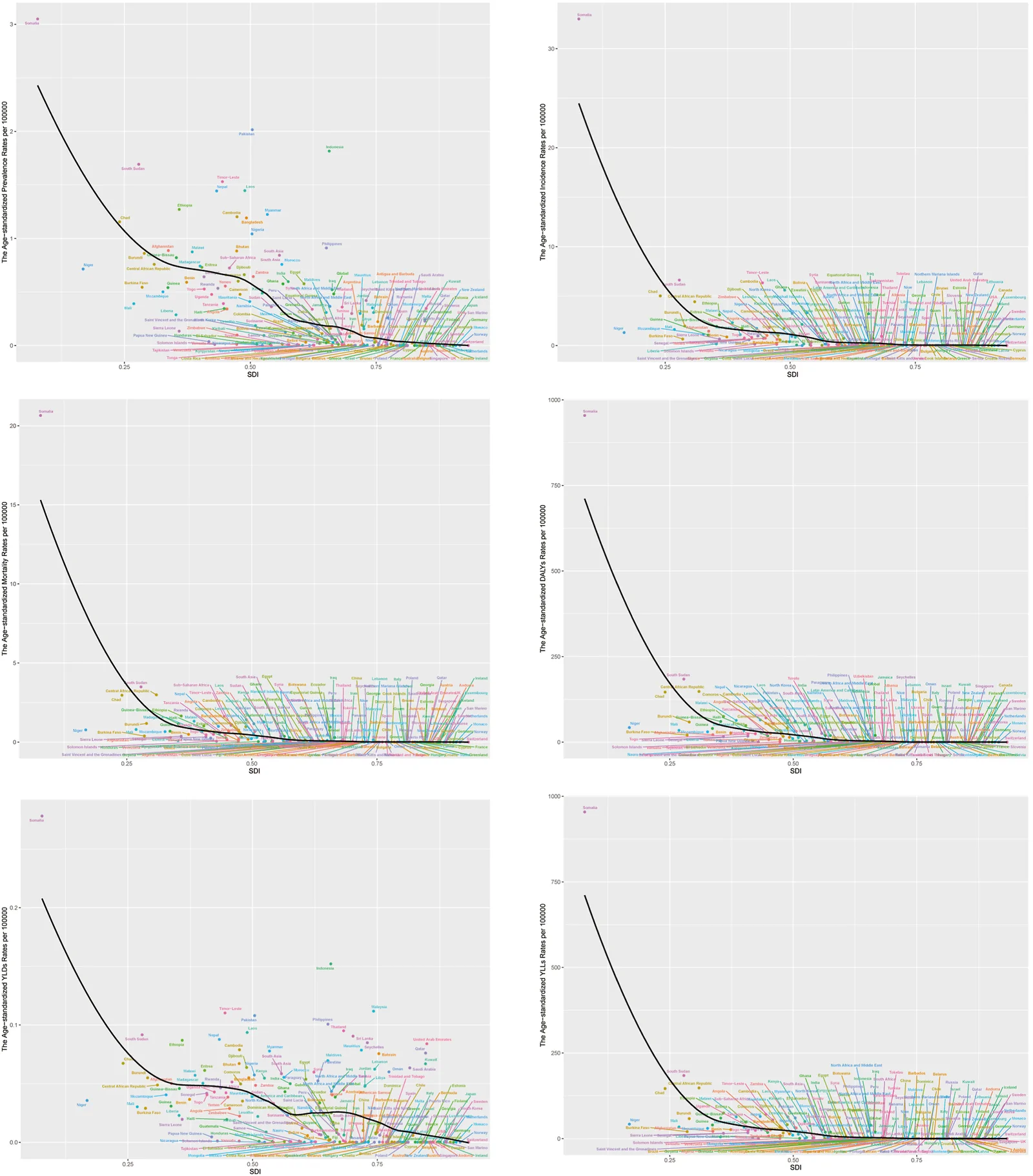
Age-standardized disease burden rates (per 100,000 population) across SDI regions. Horizontal axis: SDI value; Vertical axis: rate. SDI: socio-demographic index; DALYs: disability-adjusted life years; YLDs: years lived with disability; YLLs: years of life lost.

In 2021, regions with a high burden of global tetanus were primarily concentrated in sub-Saharan Africa, South Asia, and Southeast Asia, with disease burden indicators depicted in dark red on the map. In contrast, light blue regions such as Europe, the Americas, Oceania, and northern Asia have reduced the health burden caused by tetanus to relatively low levels.

### 3.3 Decomposition analysis

As illustrated in Fig. 5, through decomposition analysis, we examined 3 contributing components: aging (the elderly population), epidemiological changes (disease prevalence, incidence, mortality, and related interventions), and demographic changes (the absolute number of the population). Fig. 5 reveals that all disease burden factors exhibit negative values, indicating that changes in epidemiological factors (blue) are the primary drivers behind the reduction in the global burden of tetanus, while the impacts of aging and demographic changes are relatively minor.

**Fig. 5:**
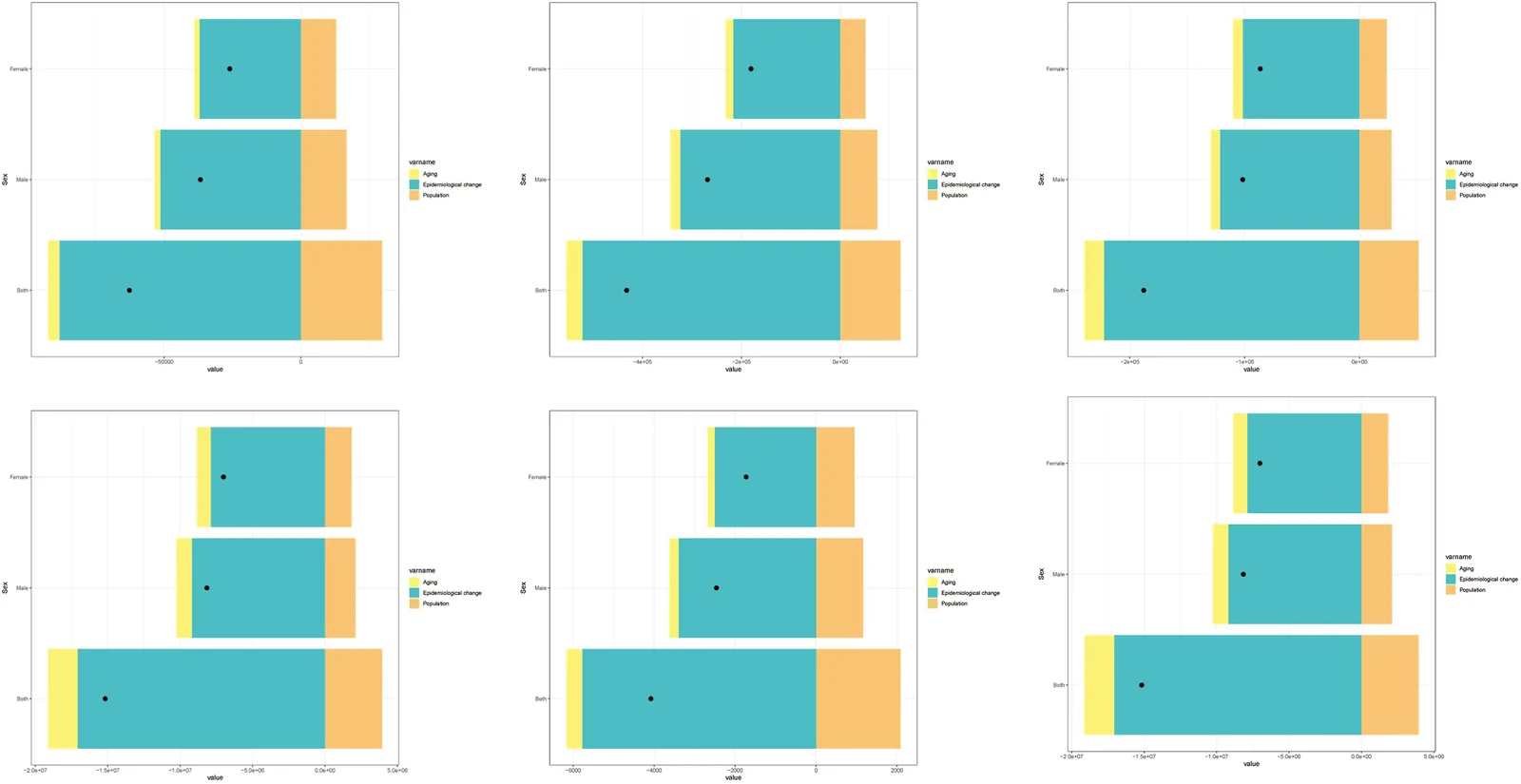
Decomposition analysis of disease burden impact by sex. Horizontal axis: values; Vertical axis: sex. DALYs: disability-adjusted life years; YLDs: years lived with disability; YLLs: years of life lost.

### 3.4 BAPC analysis

From 1990 to 2021, the prevalence, incidence, mortality, DALYs, YLDs, and YLLs due to tetanus among both males and females globally exhibited a steady declining trend, with the uncertainty intervals gradually narrowing (represented by the shaded areas in the figure). From 2021 to 2050, all indicators are projected to continue decreasing; however, the shaded area first narrows and then slightly widens, indicating a potential slight increase in uncertainty. The similarity in trend lines and shaded areas for both sexes suggests minimal sex differences (Fig. 6).

**Fig. 6:**
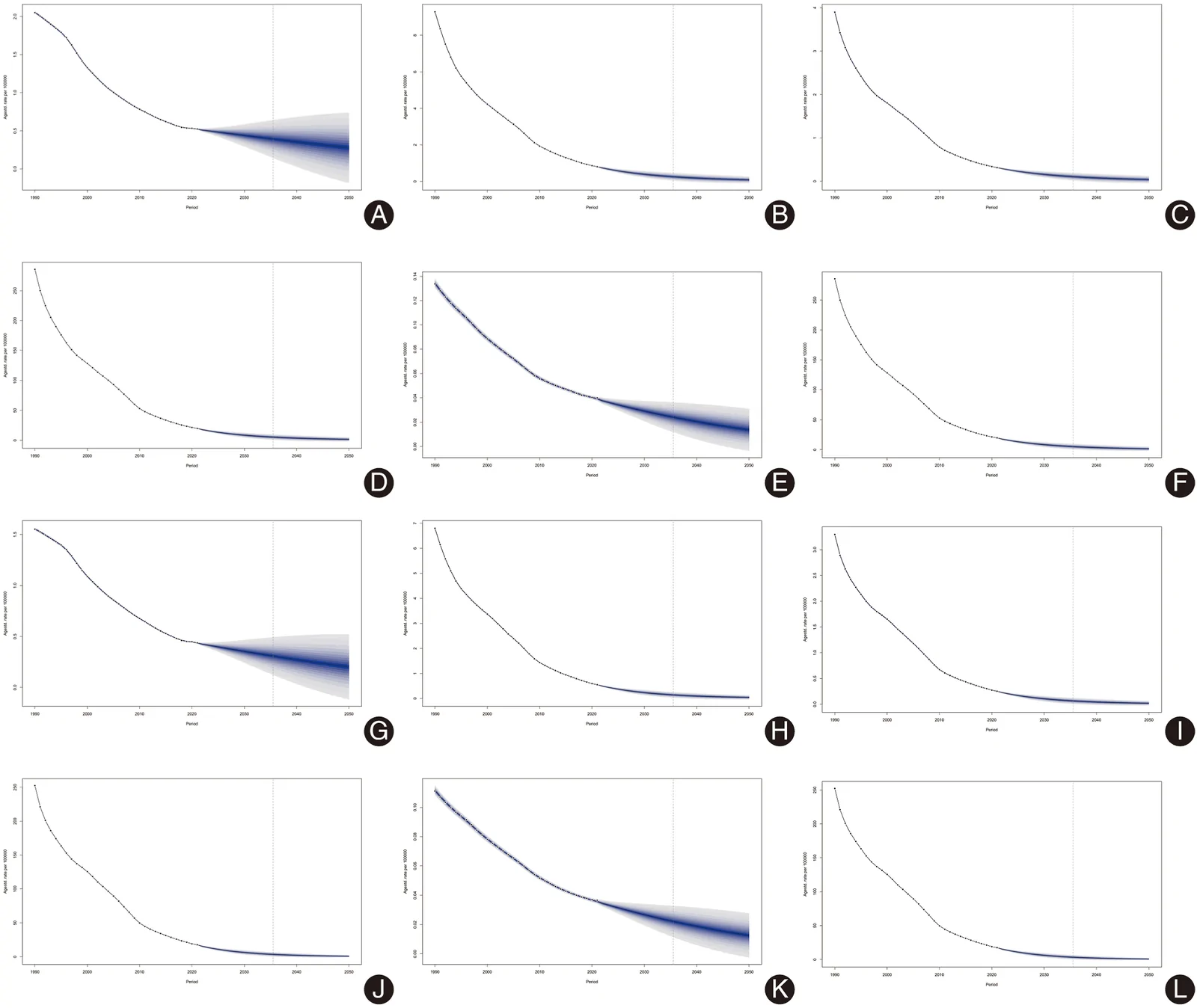
BAPC analysis. (A–F) The age-standardized prevalence rate, incidence rate, mortality rate, DALYs, YLDs, and YLLs per 100,000 males. (G–L) The age-standardized prevalence rate, incidence rate, mortality rate, DALYs, YLDs, and YLLs per 100,000 females. The shaded areas indicate the 95% uncertainty intervals. BAPC: Bayesian age-period-cohort; DALYs: disability-adjusted life years; YLDs: years lived with disability; YLLs: years of life lost; *UI*: uncertainty intervals.

## 4 Discussion

Tetanus mainly presents with spastic paralysis symptoms. Its toxin undergoes retrograde axonal transport to the spinal cord and brainstem, irreversibly binding to receptors and inhibiting inhibitory neurotransmitter release. This disrupts spinal and autonomic neuron regulation, causing muscle rigidity, painful spasms, and autonomic dysfunction.^16^, ^17^, ^18^ It is prevalent in low- and middle-income countries and, though rare in developed nations, remains challenging clinically.^5^ As early as 1948, Soviet research showed 90% of cases occurred in rural areas, linked to soil *Clostridium tetani* contamination, with preschool and school-aged children most vulnerable.^19^

Tetanus prevention mainly relies on tetanus toxoid vaccination. Since the 1970s, WHO has included tetanus immunization in national programs, giving newborns primary doses intramuscularly at 3, 4, and 5 months, a booster at 18 months, and a diphtheria-tetanus vaccine at 6 years.^20^ In 1960, the Soviet Union launched a nationwide prevention program, mandating vaccination for certain residents, children, and high-risk adults; providing prophylactic treatment for injuries; recommending vaccination for pregnant women; educating women on disease etiology; and scheduling child revaccination. This reduced the national average incidence rate by over 30%, with even greater drops in some regions.^19^ WHO recommends 3 doses of diphtheria-tetanus-pertussis vaccine in infancy, followed by boosters at 4–7 and 15 years as part of the Expanded Programme on Immunization.^21^

Prevention of neonatal tetanus relies on aseptic delivery practices, maternal immunization, umbilical cord care, and instrument sterilization; however, these methods have not been widely adopted. Demicheli et al.^22^ confirmed the effectiveness of the tetanus toxoid vaccine in preventing neonatal tetanus, emphasizing the importance of multiple doses and supporting the promotion of the vaccine in high-risk areas. Due to the widespread presence of the pathogen and the lack of natural immunity in humans, tetanus cannot be completely eradicated. Prevention and control strategies primarily focus on vaccination to eliminate the disease.^23^ Currently, the main global risk factors for tetanus include injecting drug use and diabetes, with tetanus cases in the UK being associated with heroin use.^24^ The higher disease burden among males compared to females may be related to unsafe circumcision practices in certain regions.^25^ Therefore, the global community still faces challenges in preventing tetanus.

Launched in 1991, the GBD 2021 engages over 8000 researchers and analysts from over 150 countries. Despite limitations, it remains a rigorous, standardized, ongoing project offering practical health metric references for diverse stakeholders.^26^ As research progresses, the GBD expands its assessment scope, adding etiologies, risk factors, and regions, and refining age-group analyses. It also supports predictive and extended research, like assessing antimicrobial resistance burden.^27^, ^28^, ^29^ Since 1991, the GBD has grown into a large-scale global collaborative effort, integrating scientific research across nations. It continuously optimizes data collection, analysis, and synthesis to improve transparency and cross-regional comparability of health metrics, while systematically assessing uncertainty sources. Despite limitations, the GBD provides authoritative health assessment data through dynamic updates and standardized methods, with analytical dimensions expanding to cover more diseases, risk factors, regions, and refined age groups, supporting predictive modeling and extended studies.^26^, ^27^, ^28^, ^29^, ^30^

In this study, we utilized data from GBD 2021 to analyze trends in the global burden of tetanus over the past 30 years and conducted a decomposition analysis of the epidemiological trends of tetanus worldwide. Additionally, we employed a BAPC model to predict epidemiological trends for tetanus in 2050.

This study found that, compared to 1990, the global burden of tetanus had significantly decreased by 2021. From 1990 to 2021, remarkable progress was made globally in the prevention and control of tetanus, with reductions in the number of prevalent cases, new cases, and deaths, as well as a substantial decrease in the disease's impact on quality of life, resulting in an overall shift from a heavy to a lighter burden. Nevertheless, the global burden of tetanus among newborns remained relatively high in 2021. This may be attributed to the challenges in preventing neonatal tetanus, such as the implementation of maternal immunization programs. Regarding sex disparities, the disease burden was higher among males than females, possibly linked to unsafe circumcision practices in certain regions^29^ and the lower vaccination rates among males due to the WHO's focus on maternal immunization.^25^^,^^31^ Subsequent directions for tetanus prevention should maintain current immunization policies while focusing on the newborn population (<28 days), strengthening aseptic delivery practices, maternal immunization, umbilical cord care, and instrument sterilization, as well as advocating for effective maternal immunization policies. Additionally, efforts should be made to increase public awareness about tetanus, expand the scope of awareness campaigns, and eliminate unsafe circumcision practices.

Analysis of disease burden based on different SDI levels revealed a strong negative correlation between tetanus burden and SDI levels. Although the global burden of tetanus generally showed a downward trend, significant disparities existed across regions with varying SDI levels. Areas with higher SDI levels maintained low disease burdens over the long term, while low-SDI regions continued to bear a relatively higher burden. This disparity may be attributed to the remarkable achievements in prevention and control, coupled with more comprehensive healthcare resources in high-SDI areas. In contrast, low-SDI regions faced greater challenges in prevention and control due to limited resources. These findings are consistent with those of Li et al.,^32^ who also noted that relevant data remain controversial; while the decline in tetanus cases among children and adults was relatively limited, global trends obscured significant disparities in disease burden across regions of varying income levels. Li et al.^32^ similarly pointed out that a study of hospital records in England from 2001 to 2014 revealed an underreporting rate of tetanus cases as high as 88%,^33^ raising questions about data accuracy.

Decomposition analysis results showed that epidemiological changes (disease prevalence, incidence, mortality, and interventions) were the primary factors influencing the global burden of tetanus. These findings can assist public health authorities in more precisely focusing on key factors when formulating tetanus prevention and control strategies, optimizing resource allocation, and enhancing prevention and control effectiveness. Future research could further observe prevalence, incidence, mortality, and tetanus interventions to develop more appropriate preventive measures and optimize vaccination policies, providing a basis for prevention and control efforts. Predictions of the global tetanus burden in 2050, based on the BAPC model, indicated a continued decline in the global burden over the coming decades. However, uncertainty may rise slightly, possibly due to ongoing conflicts in certain regions of the world. This underscores the necessity to continue improving access to trauma care resources and to enhance vaccination for pregnant women and children. To ensure the timeliness of prevention and control strategies, the management of tetanus sequelae should be integrated into the health system, and the BAPC model should be regularly updated with new data to optimize predictions.

This study has certain limitations. First, the GBD model lacks detailed local data worldwide and relies on model estimates, which may not accurately describe the epidemiological characteristics of tetanus worldwide or reflect structural disparities and uneven urban-rural development between countries. Second, GBD 2021 cannot extract data from the injury epidemiology perspective, and only a limited number of literature sources mention the association between tetanus and drug use or unsafe circumcision among its global tetanus causes. The GBD prediction model may not fully account for socioeconomic changes and diverse risk exposures in different countries, potentially leading to errors in predicting the global burden of tetanus. GBD 2023 incorporates updated and more comprehensive data sources, including newly conducted population baseline survey data and improved health information system data. Additionally, GBD 2023 employs more sophisticated analytical methods, including more complex statistical models and improved data processing and estimation algorithms, whereas GBD 2021 data have not undergone such updates. Therefore, future research should prioritize the use of GBD 2023. The comparability and generalizability of the results are compromised by regional population differences and temporal dynamics, which stem from limited data in the GBD database. Furthermore, the BAPC analysis prediction model relies on the assumption of a linear continuation of past trends, a limitation hindering its ability to capture nonlinear changes, rendering it overly reliant on historical data and limiting its capacity to anticipate future complex changes.

In conclusion, the global burden of disease caused by tetanus has been on a declining trajectory. This positive trend primarily stems from the widespread global coverage of tetanus vaccines, which has effectively reduced the risk of tetanus infection. Moreover, significant advancements in medical conditions have played a crucial role. The broad distribution of high-quality healthcare resources and the improved standardization and professionalization of basic medical procedures, such as wound care, have further contributed to this decline. Additionally, the growing global focus on health issues and the heightened health awareness among the public have also been instrumental in driving down the overall disease burden of tetanus. However, the demographic structure of the tetanus disease burden remains relatively high among newborns aged <28 days, with a greater burden on males than females. In future tetanus prevention efforts, it may be necessary to target tetanus immunization for newborns and pregnant women as key prevention and control populations, and further optimize prenatal check-ups and immunization procedures to ensure that every newborn receives timely and adequate tetanus vaccination. For males, it is essential to strengthen health education and expand the geographical scope of health promotion campaigns to discourage drug use and unsafe circumcision practices. It is hoped that through these targeted preventive measures, the burden of tetanus can be maintained at a low level.

## Declaration of using generative AI and AI-assisted technologies in the writing process

During the preparation of this work, the author utilized ERNIE Bot with the aim of enhancing language quality and readability. After using this tool/service, the author reviewed and edited the content as necessary and takes full responsibility for the content of the publication.

## CRediT authorship contribution statement

**Tongying Liu:** Writing – original draft, Visualization, Data curation. **Menglong Liu:** Writing – review & editing, Writing – original draft, Visualization, Data curation. **Guosheng Yang:** Writing – original draft, Data curation. **Qingqing Ye:** Writing – original draft, Data curation. **Tianjing Sun:** Writing – original draft, Data curation. **Xiaojun Zhang:** Writing – original draft, Data curation. **Anping Liu:** Writing – original draft, Visualization. **Renjie Song:** Visualization, Investigation. **Can Luo:** Visualization, Investigation. **Xiaofei Huang:** Visualization. **Anyong Yu:** Writing – review & editing, Writing – original draft, Conceptualization. **Haizhen Duan:** Writing – review & editing, Writing – original draft, Visualization, Data curation, Conceptualization.

## Ethical statement

The data for this study were sourced from the GBD database. The GBD research consortium has formulated a comprehensive set of data usage policies, which we strictly adhered to throughout the entire research process. The data in the GBD database are aggregated and anonymized, effectively protecting the privacy of individual patients. In this study, we did not access or use any personally identifiable information, eliminating potential privacy risks. We declare that there are no conflicts of interest in this study. It was conducted solely for academic and scientific purposes, without being influenced by commercial or other external factors.

## Funding

The study was funded by the National Natural Science Foundation of China - China Academy of General Technology Joint Fund for Basic Research (Funding No.: 82260385), the Science and Technology Program of Guizhou Province, China (Funding No.: Qiankehejichu-ZK [2023] 580), the Health Commission of Guizhou Province, China (Funding No.: gzwkj2022-103), the Guizhou Province Clinical Research Center for Emergency Medicine (Funding No.: LCZX[2025]004), the National Clinical Key Specialty (Funding No.: xm040212).

## Declaration of competing interest

The authors declare no competing interests.
